# A natural marmoset model of genetic generalized epilepsy

**DOI:** 10.1186/s13041-022-00901-2

**Published:** 2022-02-10

**Authors:** Xiangyu Yang, Zhitang Chen, Ziying Wang, Guang He, Zhiqiang Li, Yongyong Shi, Neng Gong, Binglei Zhao, Yifang Kuang, Eiki Takahashi, Weidong Li

**Affiliations:** 1 Bio-X Institutes, Key Laboratory for the Genetics of Development and Neuropsychiatric Disorders (Ministry of Education), Shanghai Key Laboratory of Psychotic Disorders, and Brain Science and Technology Research Center, Institute of Psychology and Behavioral Sciences, Shanghai Jiao Tong University, Shanghai, 200240 China; 2 WLA Laboratories, World Laureates Association, Shanghai, 201203 China; 3 Institute of Neuroscience, Key Laboratory of Primate Neurobiology, CAS Center for Excellence in Brain Science and Intelligence Technology, Chinese Academy of Sciences, Shanghai, 200031 China

**Keywords:** Natural marmoset model, Non-human primate, Generalized epilepsy, ECoG recording, Behavioral analysis

## Abstract

Epilepsy has been extensively studied as a common neurological disease. Efforts have been made on rodent and other animal models to reveal the pathogenic mechanisms of epilepsy and develop new drugs for treatment. However, the features of current epilepsy models cannot fully mimic different types of epilepsy in humans, hence non-human primate models of epilepsy are required. The common marmoset (*Callithrix jacchus*) is a New World monkey that is widely used to study brain function. Here, we present a natural marmoset model of generalized epilepsy. In this unique marmoset family, generalized epilepsy was successfully induced by handling operations in some individuals. We mapped the marmoset family with handling-sensitive epilepsy and found that the epileptic phenotype can be inherited. These marmosets were more sensitive to the epilepsy inducers pentylenetetrazol. Using electrocorticogram (ECoG) recordings, we detected epileptiform discharge in marmosets with a history of seizures. In summary, we report a family of marmosets with generalized seizures induced by handling operations. This epileptic marmoset family provides insights to better understand the mechanism of generalized epilepsy and helps to develop new therapeutic methods.

**Supplementary Information:**

The online version contains supplementary material available at 10.1186/s13041-022-00901-2.

Epilepsy is a common chronic brain disorder characterized by recurrent seizures that affects over 70 million people worldwide [1, 2]. A third of patients have genetic generalized epilepsies that exhibit a polygenic and heritable etiology [3]. In the past few decades, animals such as rodents have been used to investigate the mechanisms and treatments of epilepsy [4]. Due to the differences in genetic constitution and brain structure, rodent models cannot fully mimic human epilepsy. To address this issue, non-human primate models are considered suitable models of nervous system diseases because the non-human primate brain has very similar genetic, neurochemical, neurophysiologic, and structural features to human brains [5]. Killam et al. first described a non-human primate baboon model of photosensitive epilepsy in 1966, which was characterized by intermittent light stimulation (ILS)-induced seizures [6, 7]. This is the first natural epileptic non-human primate model. However, to date, no natural model of epilepsy in marmosets has been reported.

The common marmoset (*Callithrix jacchus*) is a small New World monkey that has been frequently used because of its genetic constitution, body size, and unique reproductive characteristics (twice a year to either twins or triplets) [8–10]. Genome-wide data have been obtained from common marmosets, and the results showed that most of the genes are highly conserved between marmosets and humans [11, 12]. This suggests that marmosets are a valuable biomedical model for primates. In epilepsy research, marmosets are mainly used for drug-induced epilepsy models and are reliable for the evaluation of antiepileptic drugs [13–15].

Here, we showed for the first time a marmoset family with generalized epilepsy. In this marmoset family, some individuals showed significant seizure phenotypes in response to handling stimulation. The seizure symptoms include limb convulsions, movement disorders, vomiting, and salivation, which are typical phenotype of human epileptic seizures. Moreover, we found that this phenotype was stably inherited from generation to generation. We suggest that this natural epileptic marmoset is a potential non-human primate model for understanding the mechanism of epilepsy.

Those epileptic marmosets were discovered as a result of routine handling for health checks, such as weight measurement. The first epileptic marmoset was born in 2002 by tracing the history of seizures in this family. The main branch of this epileptic marmoset family contains 51 marmosets across four generations. The incidence rate of epileptic seizures in the four generations are 2/2(G I), 15/22(G II), 8/16(G III), 3/11(G IV), respectively (Fig. 1a). Mapping the marmoset family indicated that this handling-induced seizure phenotype was stably inherited. According to morbidity and reproductive characteristics of marmoset, we speculate that this is most likely an autosomal dominant genetic disease.

**Fig. 1:**
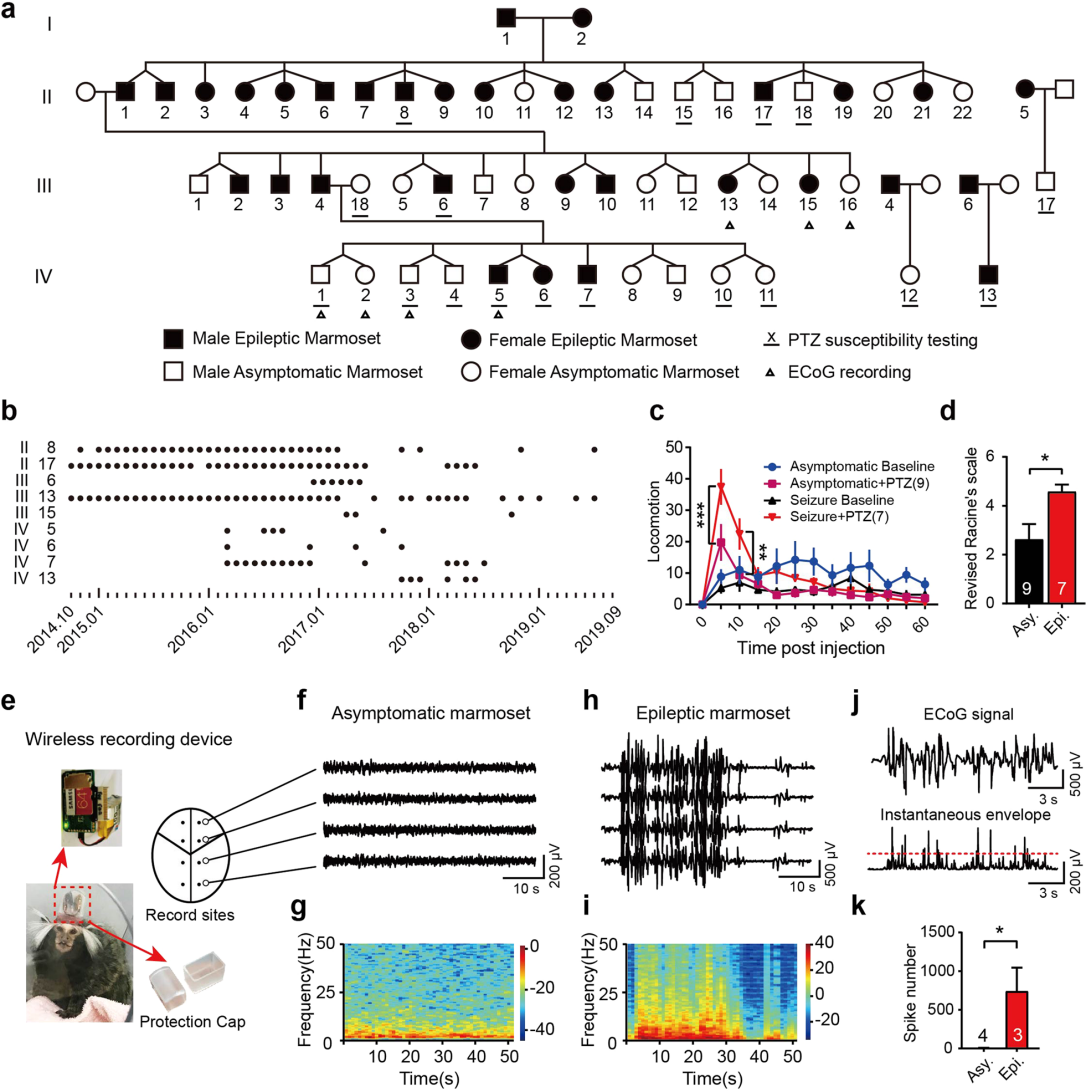
Characterization of genetic generalized epilepsy in a marmoset family. **a** Pedigree of the epileptic marmoset family. **b** Seizure records of the epileptic marmosets involved in the study. **c** Time course of locomotion after PTZ injection. **d** Seizure scores in marmosets treated with PTZ. **e** Schematic diagram of EEG recording in marmosets. **f**, **h** Typical ECoG traces after handling in asymptomatic marmosets and epileptic marmosets. **g**, **i** Spectrogram corresponding to typical ECoG traces in asymptomatic marmosets and epileptic marmosets. **j** Main procedure of epileptiform spike detect modelling. **k** Number of detected epileptiform spikes during 10 h of free-roaming. Data are presented as mean ± SEM. *P < 0.05; **P < 0.01; ***P < 0.001. *P*-value is determined by two-way ANOVA followed by Bonferroni's multiple comparisons test (**c**) or unpaired t-test (**d**, **k**)

In epileptic case, seizures were triggered by handling. The seizure started with the clonic fore limb followed by crows myoclonia and generalized tonic–clonic seizures. We found that the earliest onset of seizures in the marmoset was approximately 11 months old (Additional file 1: Table S1). Epileptic seizure records during the observation period are shown in Fig. 1b.

PTZ-induced seizure susceptibility was also assessed in the marmosets. The epileptic marmosets exhibited more severe symptoms (Fig. 1c, d). After PTZ treatment, locomotion events increased in epileptic marmosets, but decreased in asymptomatic marmosets (Additional file 1: Fig. S1a). PTZ administration also induced significant changes in other behaviors, such as scratching and head shakes/mouth cleaning behaviors (Additional file 1: Table S2 and Fig. S1 b-d). In the PTZ test, six epileptic marmosets showed clonic (IV/V) seizures, but only three asymptomatic marmosets developed clonic seizures. These results indicate that epileptic marmosets have higher sensitivity to PTZ.

We chose ECoG recording for characterization studies of brain activity. The ECoG recording pattern is shown in Fig. 1e. During recording, epileptiform whole-brain discharge was observed in all three epileptic marmosets, with a frequency of 1–6 Hz (Fig. 1h, i). In the ECoG data from free-roaming epileptic marmosets (10 h), we detected epileptic spikes using the wavelet transform method (Fig. 1j). By contrast, there were fewer epileptic spike waves in the asymptomatic marmosets than the epileptic marmosets (Fig. 1k).

In this study, we identified a family of epileptic marmosets with genetic predisposition. After a long period of retrospective investigation and observation, we tagged epileptic individuals in this family and mapped the family pedigree, and specified the behavioral characteristics of the epileptic marmosets. Epileptic marmosets reported in other studies have similar behavioral phenotypes to the marmosets we found, but researchers attributed the phenotype to viral infection [5]. Based on our observations, a genetic etiology may be the cause of epileptic seizures in this marmoset family. Hence, future studies are needed to reveal the cause of epileptic phenotypes, especially by using genetic analysis methods. We chose whole-genome sequencing to screen for candidate pathogenic genes. However, to date, no specific mutations have been found in this family.

PTZ susceptibility testing showed that epileptic marmosets produced more pronounced epileptic phenotypes, suggesting the existence of neurological functional variations. In vivo electrophysiological results showed that handling induced significant epileptic seizures but lacked synchronous behavioral phenotypes. This suggests that a more complex mechanism could be underlying behavioral seizures. At present, we have observed significant seizure discharges after handling, but more elaborate methods are needed to reveal the characteristics and mechanisms of seizure origin in the marmoset brain.

Combined with the above results, the discovery of this familial generalized epileptic marmoset will further enrich the application of non-human primate animals in the field of epilepsy research. This epileptic marmoset model is another natural epilepsy model that is different from photosensitive baboons, which is conducive to the study of the mechanism and treatment of epileptic seizures. In addition, further genetic research will clarify the genetic mechanism of this familial generalized epilepsy, which could help advance pathological studies in clinical settings.

## Supplementary Information

**Additional file 1: Table S1**. The detail information of epileptic marmosets in this study. **Table S2.** The frequencies of various behaviors in marmosets treated with PTZ **Fig. S1.** Various behaviors in marmosets treated with PTZ. **a** Total number of locomotion during the phase I (0–10 min) and phase II (11–60 min). **b** Scratching, **c** Mouth cleaning, **d** Head shakes behaviors in asymptomatic and epileptic marmosets. Data are expressed as mean ± SEM. Two-way ANOVA followed by Fisher's LSD test was used to compare behaviors between groups. *P < 0.05, **P < 0.01, ***P < 0.001.

## Data Availability

All raw data supporting the findings of this study are available upon reasonable request.

## Abbreviations

ECoG: Electrocorticogram
PTZ: Pentylenetetrazol
ILS: Intermittent light stimulation
Asy: Asymptomatic marmoset
Epi: Epileptic marmoset
