# Supplementary material for: A natural marmoset model of genetic generalized epilepsy
Source: Mol Brain. 2022 Feb 10;15:16. doi: 10.1186/s13041-022-00901-2 (PMC8832845; doi:10.1186/s13041-022-00901-2)
Supplement: Supplementary file 1 — Additional file 1: Table S1. The detail information of epileptic marmosets in this study. Table S2. The frequencies of various behaviors in marmosets treated with PTZ Fig. S1. Various behaviors in marmosets treated with PTZ. a Total number of locomotion during the phase I (0–10 min) and phase II (11–60 min). b Scratching, c Mouth cleaning, d Head shakes behaviors in asymptomatic and epileptic marmosets. Data are expressed as mean ± SEM. Two-way ANOVA followed by Fisher's LSD test was used to compare behaviors between groups. *P < 0.05, **P < 0.01, ***P < 0.001. [file 13041_2022_901_MOESM1_ESM.docx]

**Additional Materials for**

**A natural marmoset model of genetic generalized epilepsy**

**Materials and Methods**

**Animals**

All animal experiments were approved by the Institutional Animal Care and Use Committee (IACUC) of Shanghai Jiao Tong University (No.10645, Shanghai, China). Twenty-one common marmosets (*Callithrix jacchus*, 45-118 months old, 280–400 g) were reared at the CLEA Marmoset Breeding Facility (CLEA Japan Inc., Gifu, Japan) under approval (No.55-019CJ) from the Institutional Animal Care and Use Committee of CLEA Japan Inc. Marmosets were maintained on a 12-h light-dark cycle at 27°C and 50% humidity. Marmosets were allowed ad libitum access to water and food pellets (CMS-1M; CLEA Japan Inc.) with vitamins C and D, calcium, and acidophilus. Hot water and comb honey were also added to soften the pellets and improve the animals’ preference for the food. This study was performed using 21 marmosets including 12 asymptomatic and 9 epileptic marmosets (Fig. 1a-b, Table S1). We conducted the PTZ induced seizure behavioral test using 17 marmosets [10 asymptomatic (II-15, II-18, III-17, III-18, IV-1, IV-3, IV-4, IV-10, IV-11, IV-12) and 7 epileptic (II-8, II-17, III-6, IV-5, IV-6, IV-7, IV-13) marmosets]. One asymptomatic marmoset (III-18) was excluded from data analysis due to vomit asphyxia induced death during behavioral experiment. Thus, we analyzed the data and showed the results in Fig. 1c-d, Table S2, and Fig. S1 using 16 marmosets (9 asymptomatic and 7 epileptic marmosets). In the ECoG recording study, we used 7 marmosets (Asy: III-16, IV-1, IV-2, IV-3; Epi: III-13, III-15, IV-5) and the results were shown in Fig. 1f-k. The ECoG study on 4 marmosets (Asy: III-16, IV-2; Epi: III-13, III-15) was conducted without PTZ test. ECoG study on other 3 marmosets (Asy: IV-1, IV-3; Epi: IV-5) was performed 3 months after the PTZ test to allow PTZ metabolized completely.

**PTZ susceptibility testing**

The PTZ (S4587; Selleck Chemicals, Houston, TX, USA) test was conducted based on a previous study during the light phase of the cycle[1]. Each marmoset was habituated in an individual observation cage for at least 30 min prior to the PTZ test. To assess the susceptibility of PTZ-induced seizures, marmosets were injected intraperitoneally with 35 mg/kg PTZ. Before PTZ treatment, their natural behavior was monitored for 60 min. After PTZ injection, each marmoset was immediately placed in an observation cage. Seizures of marmosets were recorded for 60 min and evaluated using the revised Racine seizure scale.

**Seizure classification**

Seizure behaviors were evaluated based on the revised Racine seizure scale for marmosets proposed in a previous study[1-3]. The seizure activities of the marmosets were classified as follows:

I. Mouth cleaning-like behavior (rubbing the face along the perch and wire mesh);

II. Head clonus/shaking (rapid and violent head movement);

III. Forelimb clonus;

IV. Bilateral forelimb clonus, straub tail, postural impairment;

V. Generalized clonic seizures.

**Behavior analysis**

The behaviors were recorded using a video camera. The locomotion events were recorded by movements of the animal involving both limbs, such as walking, jumping, running, and climbing. These events were counted in 5 min intervals for 60 min, and were divided into two phases: phase I (0-10 min) and phase II (11-60 min). We also recorded the time spent on scratching, and counted the events of head shakes/mouth cleaning.

**Electrode implant surgery**

The implantation protocol here was modified from the previously described protocol[4]. All surgical procedures were performed in a dedicated surgical room under sterile conditions. Briefly, marmosets were deeply anesthetized with intramuscular injection of ketamine (30 mg/kg, Daiichi Sankyo Propharma Co., Ltd., Tokyo, Japan), xylazine (2.5 mg/kg, Bayer AG, Leverkusen, Germany). Marmoset scalp and underlying muscle are excised from the top of the skull. A dental drill was used to create holes on each implantation site. Eight ECoG electrodes (Jiangsu Braintech, China) were separately implanted bilaterally into the frontal cortex (AP + 9.0 mm, ML ± 5 mm), motor and premotor cortex (AP + 4.0 mm, ML ± 5 mm), parietal cortex (AP -3.0 mm, ML ± 5 mm) and occipital cortex (AP-10 mm, ML ± 5 mm). The electrodes were affixed to the skull using dental cement. After electrode implantation, a protective cap baseplate was fixed to the skull using dental cement, and the electrode connector was covered with a protective cap. Marmosets were administered analgesic (Meloxicam, Towa Pharmaceutical Co., Ltd, Tokyo, Japan) and anti-inflammatory agents (Penicillin G potassium, Meiji Co., Ltd., Tokyo, Japan). Marmosets were allowed to recover from surgery for 2 weeks.

**ECoG data collection and analysis in free roaming marmoset**

The marmosets were allowed to freely roam in the recording cage. An 8-channel neural recording device (NeuroAir 1.0, Jiangsu BrainTech, China) was connected to the electrodes. The protection cap was removed from the base of the cap. ECoG signals were recorded during the light phase of the cycle. ECoG data from 10 am to 8 pm were extracted for analysis, with a total duration of 10 h. The raw data were down-sampled at 500 Hz, and a notch filter with 60 Hz was used to remove power frequency interference during data acquisition. Data were baseline corrected by subtracting the mean of all channels, re-referenced to the average of all channels, and digitally filtered offline at 0.5-200 Hz.

**Epileptic spike detection**

The wavelet transform has multi-resolution characteristics, and a more accurate signal can be observed. By properly selecting the wavelet basis function, the wavelet transform can characterize the local characteristics of the signal in both the time and frequency domains. According to the non-stationary characteristics of ECoG signals, data of the same duration were selected for the wavelet transform. Because the cross-correlation value of the db4 function and the epilepsy signal is the largest, db4 was selected as the wavelet basis function in this study, and a 7-level-decomposition transform is used to decompose the signal, namely the detail coefficients D1, D2, D3, D4, D5, D6, D7, and the approximate coefficient A7 [5]. By setting a threshold to filter out maxima points caused by noise and other small interferences, the number of coefficients of each layer is higher than the threshold, that is, the number of spikes is counted. The spike numbers of different phenotypes of the marmosets were compared and analyzed.

**Statistical analysis**

Data were analyzed using GraphPad Prism software ver.6 (GraphPad, CA, USA). In all experiments, the experimenters were blinded to the group and treatment of the marmosets. The data were analyzed using Student's t-test or two-way ANOVA, followed by Fisher's LSD tests/ Bonferroni's test for multiple group comparisons. Values are presented as the mean ± standard error of the mean (SEM). Statistical significance was set at *P* < 0.05.

**References**

1. Bachiega JC, Blanco MM, Perez-Mendes P, Cinini SM, Covolan LMello LE. Behavioral characterization of pentylenetetrazol-induced seizures in the marmoset. Epilepsy Behav. 2008; *13*: 70-76.

2. Ishikawa A, Mizuno Y, Sakai K, Maki T, Tanaka R, Oda Y, Niimi KTakahashi E. Kainic acid-induced seizures in the common marmoset. Biochem Biophys Res Commun. 2020.

3. Fisher RS, Cross JH, French JA, Higurashi N, Hirsch E, Jansen FE, Lagae L, Moshé SL, Peltola JRoulet Perez E. Operational classification of seizure types by the International League Against Epilepsy: Position Paper of the ILAE Commission for Classification and Terminology. Epilepsia. 2017; *58*: 522-530.

4. Roy SWang X. Wireless multi-channel single unit recording in freely moving and vocalizing primates. J Neurosci Methods. 2012; *203*: 28-40.

5. Tzimourta K, Tzallas A, Giannakeas N, Astrakas L, Tsalikakis D, Tsipouras M. Epileptic seizures classification based on long-term EEG signal wavelet analysis. In International Conference on Biomedical and Health Informatics. Springer, 2017. p. 165-169.

**Table S1** The detail information of epileptic marmosets in this study.

| Epileptic marmoset ID | DOB | Age (Months) | Gender | Start Recording | Seizure Start (month age) | Number of Seizures |
| --- | --- | --- | --- | --- | --- | --- |
| II 8 | 2011.09 | 98 | M | 2014.10 | - | 32 |
| II 17 | 2013.05 | 78 | M | 2014.10 | - | 33 |
| III 6 | 2009.05 | 126 | M | 2014.10 | - | 6 |
| III 13 | 2013.06 | 77 | F | 2014.10 | - | 43 |
| III 15 | 2013.11 | 69 | F | 2014.10 | - | 3 |
| IV 5 | 2014.11 | 60 | M | 2014.12 | 2016.03(16) | 5 |
| IV 6 | 2014.11 | 56 | F | 2014.12 | 2016.03(16) | 4 |
| IV 7 | 2015.04 | 55 | M | 2015.05 | 2016.03(11) | 15 |
| IV 13 | 2015.02 | 57 | M | 2015.03 | 2017.10(32) | 6 |

All information in this table was collected from Oct. 2014 to Sep. 2019.

**Table S2** The frequencies of various behaviors in marmosets treated with PTZ

|  | **Asymptomatic marmoset (n=9)** | | **Epileptic marmoset (n=7)** | |
| --- | --- | --- | --- | --- |
|  | Baseline | PTZ | Baseline | PTZ |
| **Natural behaviors** |  |  |  |  |
| Locomotion | 124.3±40.5 | 64.2±13.5 | 59.7±17.9 | 112.7±17.3 |
| Scratching (s) | 32.7±8.1 | 6.1±2.3 | 19.3±3.4 | 3.6±0.5 |
| **Early convulsive behaviors** |  |  |  |  |
| Mouth cleaning (number of events) | 4.4±0.5 | 12.6±2.4 | 3.7±0.6 | 23.9±2.8 |
| Head clonus/shakes (number of events) | 4.7±0.6 | 10.6±1.2 | 4.9±0.8 | 18.1±1.6 |

Each animal was observed for 60 min before (baseline) and after PTZ injection.


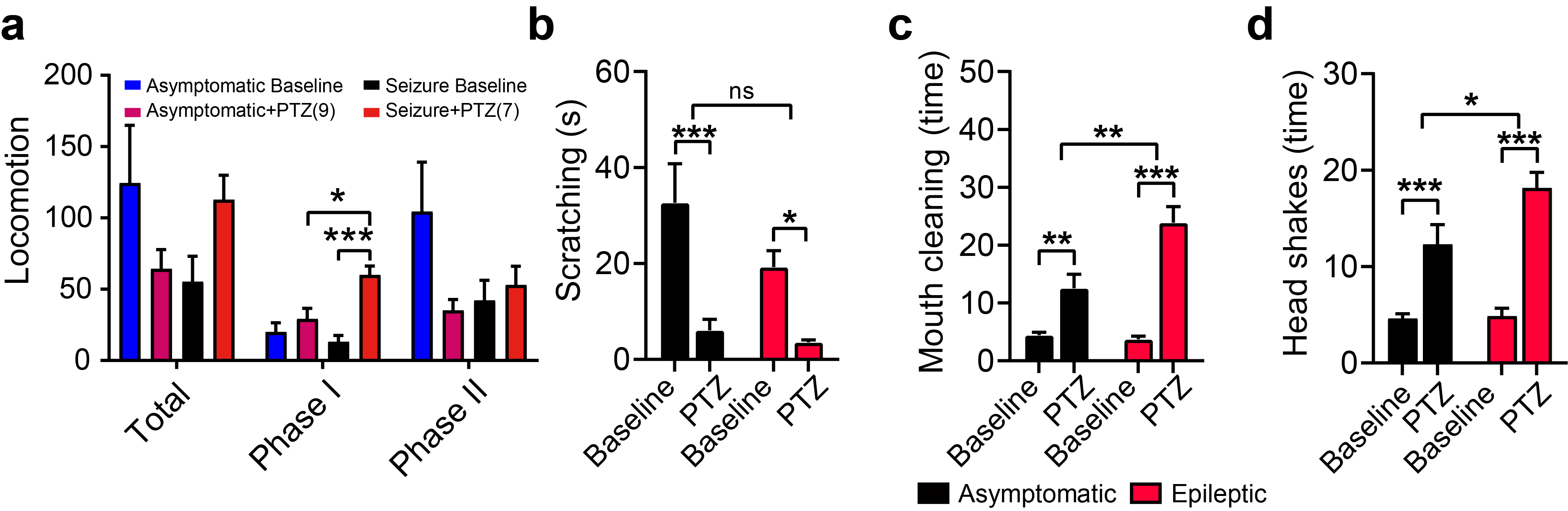


**Fig. S1.** Various behaviors in marmosets treated with PTZ. **a** Total number of locomotion during the phase I (0–10 min) and phase II (11–60 min). **b** Scratching, **c** Mouth cleaning, **d** Head shakes behaviors in asymptomatic and epileptic marmosets. Data are expressed as mean ± SEM. Two-way ANOVA followed by Fisher's LSD test was used to compare behaviors between groups. **P* < 0.05, ***P* < 0.01, ****P* < 0.001.
